# Identification of a κ-opioid agonist as a potent and selective lead for drug development against human African trypanosomiasis

**DOI:** 10.1016/j.bcp.2010.07.038

**Published:** 2010-11-15

**Authors:** Deuan C. Jones, Irene Hallyburton, Laste Stojanovski, Kevin D. Read, Julie A. Frearson, Alan H. Fairlamb

**Affiliations:** Division of Biological Chemistry & Drug Discovery, College of Life Sciences, University of Dundee, Dow Street, Dundee DD1 5EH, UK

**Keywords:** Phenotypic screening, African trypanosomiasis, Target identification, Target validation, U50,488

## Abstract

A resazurin-based cell viability assay was developed for phenotypic screening of the LOPAC 1280 ‘library of pharmacologically active compounds’ against bloodstream forms of *Trypanosoma brucei in vitro* identifying 33 compounds with EC_50_ values <1 μM. Counter-screening vs. normal diploid human fibroblasts (MRC5 cells) was used to rank these hits for selectivity, with the most potent (<70 nM) and selective (>700-fold) compounds being suramin and pentamidine. These are well-known antitrypanosomal drugs which demonstrate the robustness of the resazurin cell viability assay. The most selective novel inhibitor was (+)-*trans*-(1*R*,2*R*)-U50,488 having an EC_50_ value of 60 nM against *T. brucei* and 270-fold selectivity over human fibroblasts. Interestingly, (−)-U50,488, a known CNS-active κ-opioid receptor agonist and other structurally related compounds were >70-fold less active or inactive, as were several μ- and κ-opioid antagonists. Although (+)-U50,488 was well tolerated by the oral route and displayed good pharmaceutical properties, including high brain penetration, the compound was not curative in the mouse model of infection. Nonetheless, the divergence of antinociceptive and antitrypanosomal activity represents a promising start point for further exploratory chemistry. Bioinformatic studies did not reveal any obvious candidate opioid receptors and the target of this cytostatic compound is unknown. Among the other potent, but less selective screening hits were compound classes with activity against protein kinases, topoisomerases, tubulin, as well as DNA and energy metabolism.

## Graphical abstract

Phenotypic screening of the LOPAC library identified several potent and selective inhibitors of African trypanosomes. The κ-opioid agonist (+)-U50,488 represents a novel lead for drug discovery against sleeping sickness.

## 1 Introduction

Human African trypanosomiasis (HAT) is a disease endemic to the sub-Saharan region of Africa and is caused by two subspecies of the protozoan parasite *Trypanosoma brucei*. *T. b. gambiense* is responsible for the chronic form of the disease found in western and central Africa, accounting for over 90% of reported cases of the disease, whereas *T. b. rhodesiense* is responsible for the more acute form of the disease located in eastern Africa [1]. Only 10–15% of the 60 million people at risk of the disease are under surveillance [1] and the death rate is currently estimated at 30 000 per annum [2]. *T. b. brucei* and other *Trypanosoma* spp. are responsible for related veterinary diseases of economic importance, such as nagana in cattle.

Of the four drugs that are currently registered for use against HAT, pentamidine and suramin are used against the early stage of the disease; and melarsoprol and eflornithine (difluoromethylornithine, DFMO) are used against the late stage of the disease, when the infection has spread to the central nervous system (CNS). These treatments are beset with problems such as difficulties in administration (none are given orally), cost, duration of treatment, toxicity and resistance [3]. Melarsoprol treatment is highly toxic and responsible for iatrogenic deaths in 5% of patients. Eflornithine therapy is only effective against *T. b. gambiense* infections and presents severe economic and logistical problems in resource poor settings due to the need for 4 daily intravenous infusions over 14 days [4]. A recent clinical trial suggests the duration and frequency of treatment can be reduced by combination of eflornithine with nifurtimox [5], which may act as an interim solution until better and safer drugs are developed. The recent failure of the first orally active first stage drug, pafuramidine (DB289) [6], and rumours of increasing failures with eflornithine therapy underlines the urgent need for novel therapeutics.

Whilst many dominant paradigms of drug discovery focus on screening against molecular targets [7], there has been a resurgence of interest in phenotypic screening against whole parasites [8]. Phenotypic screening, particularly when in combination with a mammalian counter-screen, has the distinct advantage of addressing key druggability and toxicity issues early in drug discovery, thereby reducing attrition at later stages in development. In some cases phenotypic screening may identify novel molecular targets thereby accelerating drug development. However, understanding the mode of action of phenotypic screening hits can prove challenging since many drugs act by modulation of multiple intracellular targets (“network pharmacology”) [9]. Hit identification for novel targets is best approached with a screening library of diverse chemical space, but whole cell assays typically have a much lower throughput than molecular-target-based screens. Hence, we chose to screen a library of known pharmacologically active compounds against *T. brucei* cultured *in vitro* and to counter-screen actives against a human fibroblast cell line (MRC5 cells) to eliminate non-selective inhibitors. Potent and selective hits from such an approach can present exploitable shortcuts, particularly if they have already been used in humans with known dosing and toxicity information. Developing these hits could represent a low-risk, low-cost strategy for tackling orphan diseases of the poor [7].

The Library of Pharmacologically Active Compounds from Sigma–Aldrich (LOPAC 1280; international version) contains 1268 compounds that are ligands for many enzymes, receptors and ion channels in other organisms. Many are drug-like molecules and some are CNS active—an important consideration when seeking a replacement therapy for late-stage trypanosomiasis. Here we report over 30 compounds with EC_50_ values against *T. brucei* less than 1 μM, a concentration that should be readily achieved in plasma. Of these, one third have >20-fold selectivity with the κ-opioid receptor agonist U50,488 showing the greatest potency and selectivity. Some preliminary structure–activity relationships of CNS-active μ- and κ-opioid receptor agonists and antagonists are reported.

## 2 Materials and methods

### 2.1 Chemicals and materials

The LOPAC 1280 library (international version) was purchased from Sigma–Aldrich (Gillingham, UK). Pentamidine isethionate was obtained from Research Biochemicals International, eflornithine was a gift from Merrell Dow Research Institute (Ohio, USA) and melarsoprol a gift from Rhone-Poulenc (France). U69593, U5449A and naloxone were obtained from Alexxis Biochemicals (Nottingham, UK). Norbinaltorphimine, (−)-U50,488, (+)-U50,488, naltrexone, DIPPA (2-(3,4-dichlorophenyl)-*N*-methyl-*N*-[(1*S*)-1-(3-isothiocyanatophenyl)-2-(1-pyrrolidinyl)ethyl]acetamide hydrochloride) and 4-P-PDOT (cis-4-phenyl-2-propionamidotetralin) were obtained from Tocris Bioscience (Avonmouth, UK). DMSO was purchased from VWR international and HPLC-grade methanol and acetonitrile from Fluka. Resazurin, thioglycerol and PEG400 were obtained from Sigma–Aldrich (Gillingham, UK). Sterile 96-well plates were obtained from Greiner Bio-one (Stonehouse, UK).

### 2.2 Trypanosome culture

Bloodstream-form *T. b. brucei* cells (strain 427, ‘single marker’) were grown at 37 °C and 5% CO_2_ in a modified HMI9 medium [10] (HMI9-T where 0.2 mM 2-mercaptoethanol was replaced with 0.056 mM thioglycerol). Stock cultures were maintained in T75 vented cap culture flasks (Greiner, Stonehouse, UK) and subcultured every 2–3 days by 500-fold dilution into fresh medium. For microtitre plate assays, cells were counted using a Casy cell counter TT (Sharfe systems) and diluted appropriately.

### 2.3 Mammalian cell culture

Normal human MRC5 cells (diploid foetal lung fibroblasts) were used as a counter-screen for non-selective inhibitors. Stabilates were obtained from the European Collection of Cell Cultures (ECACC) were grown at 37 °C and 5% CO_2_ in a humidified incubator in Eagle's Minimal Essential Medium (Sigma–Aldrich, Gillingham, UK) supplemented with 10% foetal bovine serum (Invitrogen, Paisley, UK). Stock cultures were maintained in T75 vented cap culture flasks with half the medium changed each day. Cells were split once confluent as follows: medium was removed and the monolayer washed with PBS (137 mM NaCl, 2.7 mM KCl, 10 mM Na_2_HPO_4_, 2 mM KH_2_PO_4_, pH 7.4). Cells were detached by incubation for 2 min with 1 ml of trypsin EDTA solution (0.5 mg ml^−1^ trypsin, 0.2 mg ml^−1^ EDTA·4Na in Hanks balanced salt solution supplied by Invitrogen, Paisley, UK). Detachment was confirmed by microscopy. Fresh medium was added to the cells to subculture into new flasks at 2- to 4-fold the original volume as required.

### 2.4 Linearity of assay

*T. brucei* bloodstream-form cells and human fibroblasts were pipetted into 96-well plates to give a range of densities in a total volume of 200 μl culture medium. Resazurin (20 μl of a 500 μM stock; 45.5 μM final concentration) was added immediately to *T. brucei* cells or the next day for human fibroblasts, following incubation overnight to allow attachment to the plastic plates. Fluorescence was then measured at intervals up to 5 h (excitation of 528 nm and emission of 590 nm) on a Biotec Instruments FLX 800 fluorescent plate reader.

### 2.5 DMSO tolerance of the assay

DMSO was serially diluted across a 96-well plate in 200 μl of the appropriate medium. For *T. brucei*, 100 μl aliquots were then transferred to a plate containing 100 μl cells at 2 × 10^3^ ml^−1^. Human fibroblasts were plated at 2 × 10^4^ ml^−1^ in 100 μl of medium and incubated overnight to allow cells to adhere before the addition of medium containing DMSO (100 μl) over a range of concentrations. Parasites and mammalian cells were then incubated for 3 days, after which 20 μl 500-μM resazurin was added to each well. Plates were incubated for a further 4 h before measuring fluorescence as above.

### 2.6 EC_50_ determinations against *T. brucei*

Test compounds were dissolved in DMSO at 20 or 10 mM except for eflornithine which was dissolved in water and sterilised by filtration. The control drug (pentamidine) was dissolved in DMSO at 100 μM. HMI9-T medium (148.5 μl) was added to column 2 (B2-G2) of a sterile 96-well culture plate. HMI9-T + 1% DMSO (100 μl) was added to all remaining wells. Test compound solutions (1.5 μl) were added to column 2 (B2-G2). Pentamidine was placed on row G of all plates as a control. Threefold serial dilutions were carried out by transferring 50 μl from column 2 to the adjacent column (100 μl). The process was repeated up to column 10. HMI9-T containing 2 × 10^3^ trypanosomes ml^−1^ (100 μl) was added to all wells except column 1. HMI9-T (100 μl) was added to column 1. Columns 1 and 11 served as controls without cells and without test compound, respectively. Cells were incubated for 3 days, after which 20 μl 0.5 mM resazurin was added to each well, before measuring fluorescence after 4 h incubation. Data were processed using GRAFIT (version 5.0.4; Erithacus software) and fitted to a 3-parameter equation, where the data are corrected for background fluorescence, to obtain the effective concentration inhibiting growth by 50% (EC_50_):

(1) $$y=\frac{y_{\text{max}}}{1+{\left(i/\text{E}{\text{C}}_{50}\right)}^{s}}$$

where *y*_max_ is the uninhibited fluorescence value, *i* is the inhibitor concentration and *s* is the Hill slope of the curve.

Measurements for each compound were carried out on 3 separate occasions and the mean weighted to the standard error calculated using the following formulas, where ‘*a*’ is the standard error of EC_50_ determination ‘*A*’, etc.

(2a) $$\text{weighted mean}=\begin{array}{c} \underline{\left(A/a^{2})+(B/b^{2})+(C/c^{2}\right)}\\ \left(1/a^{2})+(1/b^{2})+(1/c^{2}\right) \end{array}$$

and

(2b) $$\text{error}=\begin{array}{c} \underline{\left(1/a)+(1/b)+(1/c\right)}\\ \left(1/a^{2})+(1/b^{2})+(1/c^{2}\right) \end{array}$$

### 2.7 Production of LOPAC daughter plates

Latch-racks containing the LOPAC library at 10 mM dissolved in DMSO were stored frozen at −20 °C. Racks were thawed at room temperature for 3 h and centrifuged in a Beckman CS-15R centrifuge at 1100 × *g* for 2 min to return any condensation on the lids to the solution. Racks were then mixed vigorously for 5 min on a platform shaker. Aliquots (50 μl) of each rack were transferred to v-bottomed polypropylene 96-well plates (Matrix) using a Janus liquid handler (PerkinElmer) to create a primary daughter set. DMSO (98 μl) was plated out to columns 2–11 of fresh v-bottom polypropylene 96-well plates using a Precision 200 liquid handler (Biotek). An aliquot (2 μl) of the primary daughter set was transferred to the DMSO-containing plates using a Platemate 2X2 liquid handler (Matrix) to create secondary daughter sets at 200 μM. At this stage, 50 μl of 200 μM pentamidine was added to wells E1–H1, 50 μl DMSO was added to the remaining wells of columns 1 and 12 for use as control wells. Duplicate daughter sets (set A and set B) created in this way were used for two independent screening runs to control for errors in liquid handling. Daughter sets were sealed using a nitrogen-flushing heat sealer (K Biosystems) and stored at −20 °C.

### 2.8 Screening of the LOPAC library against *T. brucei*

Daughter sets were thawed and mixed thoroughly, before 1 μl was transferred to an empty 96-well tissue-culture plate (Greiner) using a Platemate 2X2 liquid handler (Matrix). Plates were transferred to a tissue culture hood and 180 μl medium was added to each well using a WellMate dispenser (Matrix). HMI9-T (19 μl) was added to wells A1–D1 and 19 μl medium containing *T. brucei* (1.05 × 10^4^ ml^−1^) was added to all other wells using a Imact2 pipettor (Matrix). Wells A1–D1 served as background (no cell) controls; wells E1–H1 served as pentamidine controls (1 μM); and column 12 served as a full signal control (cells, no compound). Plates were incubated for 3 days after which 20 μl 0.5 mM resazurin was added to each well. Plates were incubated for a further 4 h before measuring fluorescence as above. Fluorescence signal for each well was background subtracted and expressed as percentage of the full signal controls. *Z*-prime (*Z*′) values were calculated for all plates using the following equation [11]:

(3) $$Z^{\prime }=1-\begin{array}{c} \underline{3\sigma (\text{full signal})+3\sigma (\text{background signal})}\\ \mu (\text{full signal})-\mu (\text{background signal}) \end{array}$$

where *μ* and *σ* represent the means and standard deviations, respectively

### 2.9 EC_50_ determinations against mammalian cells

Compounds with submicromolar EC_50_ values against *T. brucei* were tested against human fibroblasts as follows. Cells were detached, counted as described above and diluted to 2 × 10^4^ ml^−1^. Cells (100 μl) were added to columns 1–11 and 100 μl medium was added to column 12 of a 96-well plate. The plate was then incubated for 24 h to allow attachment. Test compounds and Doxorubicin as a control, were serially diluted in DMSO in a 96-well polypropylene v-bottomed plate using a Janus liquid handler (PerkinElmer). Samples from the test compound dilution series and QC plates (2 μl) were transferred to duplicate tissue culture 96-well plates using a Platemate 2X2 liquid handler (Matrix). These plates were transferred to a tissue culture hood and 200 μl medium added to all wells. From these plates, 100 μl was transferred to the cell-containing plates set up the previous day. Plates were incubated for 3 days after which 20 μl 0.5 mM resazurin was added to each well. Plates were incubated for a further 4 h before measuring fluorescence as above. Fluorescence signal for each well was background subtracted and expressed as percentage inhibition of the full signal control wells. EC_50_ curve fitting employed a 4 Parameter Logistic dose response curve using IDBS XLFit 4.2 Model 205:

(4) $$y=A+\frac{B-A}{1+{\left(C/x\right)}^{D}}$$

where *A* is the minimum *y*-value, *B* is the maximum *y*-value, *C* is the EC_50_, and *D* is the Hill slope. All test compound curves had floating maximum and minimum and pre-fit was used for all 4 parameters.

### 2.10 Homology searching and protein alignments

Sequences for κ-opioid receptor protein were retrieved from the National Centre for Biotechnology Information (http://www.ncbi.nlm.nih.gov/) for sheep (accession AAY66995), zebrafish (accession NP_878306), rough skinned newt (accession AAU15126), mouse (accession NP_035141 and AAA56759) and guinea pig (accession AAA67171). Global alignments were carried out using ClustalW and *T. brucei* GeneDB was searched using the TBLASTN algorithm [12].

### 2.11 Effect of (+)-U50,488 on cell growth

Flasks of *T. brucei* were seeded at 1 × 10^4^ cells ml^−1^ and incubated in the presence of the known inhibitors pentamidine and eflornithine, and the LOPAC hit (+)-U50,488 for 3 days. Flasks were set up in triplicate for each inhibitor at multiples of EC_50_ along with 3 control flasks with no inhibitor added. Pentamidine and (+)-U50,488 were added in DMSO in a volume of 0.1% of the culture. Eflornithine was dissolved directly in HMI9-T medium and sterilised by 0.22 μM filtration before diluting into the cell culture. Cell densities were determined at intervals using a haemocytometer and generation times calculated using GraFit (version 5.0.13; Erithacus software) using the following equation:

(5) $$N=N_{0}2^{t/g}$$

where *N*_0_ and *N* are the number of cells at time zero and time *t*, respectively, and *g* is the time per generation. For low cell densities, samples were concentrated 150-fold by centrifugation and resuspension in an appropriate volume of medium.

### 2.12 DMPK and efficacy studies

All animal experiments were carried out following local ethical review and under UK regulatory licensing in accordance with the European Communities Council Directive (86/609/EEC). NMRI outbred mice were purchased from Harlan laboratories, UK.

In brief, a 96-well equilibrium dialysis apparatus (HT Dialysis LLC, Gales Ferry, CT) was used to determine the free fraction in plasma for (+)-U50,488. Membranes (12–14 kDa cut-off) were conditioned in deionised water for 60 min, followed by conditioning in 80:20 deionised water:ethanol for 20 min, and then rinsed in isotonic buffer before use. Frozen female mouse plasma was thawed, centrifuged (2100 × *g*, 10 min), spiked with (+)-U50,488 (10 μg g^−1^), and 150-μl aliquots (*n* = 6 replicate determinations) loaded into the 96-well equilibrium dialysis plate. Dialysis against isotonic buffer (150 μl) was carried out for 5 h at 37 °C using an orbital microplate shaker at 125 revolutions min^−1^. At the end of the incubation period, aliquots of plasma or buffer were transferred to Micronic tubes (Micronic B.V., the Netherlands) and the composition in each tube balanced with control fluid, such that the volume of buffer to plasma is the same. Sample extraction was performed by the addition of 400 μl of acetonitrile containing a structural analogue of (+)-U50,488 as internal standard. Samples were allowed to mix for 1 min and then centrifuged at 2100 × *g* in 96-well blocks for 10 min. All samples were analysed by UPLC/MS/MS on a Quattro Premier XE Mass Spectrometer (Waters Corporation, USA) and the unbound fraction determined as the ratio of the peak area in buffer to that in plasma.

To determine brain penetration, (+)-U50,488 was administered intravenously to female NMRI mice (*n* = 3) at a dose level of 2 mg kg^−1^ (freshly prepared in 5% DMSO, v/v, in sterile saline). A blood sample (50 μl) was collected by cardiac puncture from each animal into Micronic tubes containing deionised water (100 μl) at 5 min post-dose and the brain of each animal removed into tared Covaris glass tubes. Blood and brains were stored at −80 °C until analysis.

To determine exposure, (+)-U50,488 was administered by oral gavage using a 20-gauge feeding tube fitted with a 1-ml syringe to female NMRI mice (*n* = 3) at a dose level of 150 mg kg^−1^ (freshly prepared in DMSO, PEG400, milliQ water (1:8:11, v/v, respectively)). Blood samples (10 μl) were collected from the tail vein of each animal into Micronic tubes containing deionised water (20 μl) at 0.25, 0.5, 1, 2, 4, 6 and 8 h post-dose and stored at −80 °C until analysis.

For analysis, brain tissue was homogenised in 50% (v/v) methanol in deionised water (1:2, w/v) using a Covaris S2 (K Biosciences, Hoddesdon, UK). Extraction of blood and brain samples was then performed by a method based on protein precipitation using acetonitrile containing a structural analogue of (+)-U50,488 as an internal standard. Levels of (+)-U50,488 were determined in these extracted samples by UPLC–MS/MS on a Quattro Premier XE mass spectrometer (Waters Corporation, USA). A calibration curve was constructed in blood and brain homogenate to cover at least 3 orders of magnitude for (+)-U50,488 (range 2–2000 ng ml^−1^).

For the efficacy study, groups of three female NMRI mice were infected with the bloodstream form of *T. b. brucei* (strain 427) by a single intraperitoneal injection of 10^4^ parasites in 0.2 ml HM19-T medium. Three days following infection, mice were either dosed by oral gavage with (+)-U50,488 (150 mg kg^−1^, freshly prepared in DMSO, PEG400, milliQ water (1:8:11, v/v, respectively)) or left untreated. Dosing of (+)-U50,488 treated mice was then repeated in an identical manner every 4 h for a further 32 h. Thereafter, animals were inspected daily for clinical signs of infection and wet smears of tail blood were examined microscopically as appropriate. Parasitaemia was determined using a Neubauer haemocytometer and mice were monitored for 30 days. Mice that exceeded a parasitaemia >10^8^ ml^−1^ were humanely killed since prior experience indicated that animals would succumb to an overwhelming infection by the following day.

## 3 Results

### 3.1 Linearity of the resazurin cell viability assays

**Fig. 1:**
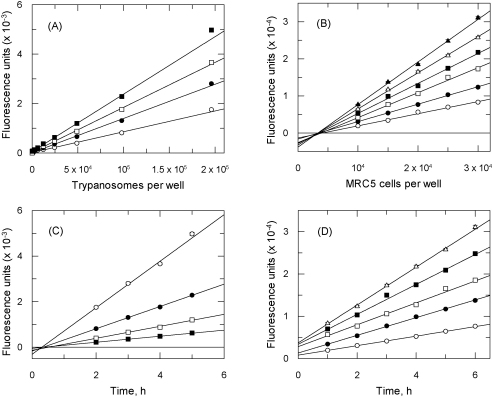
Linearity of the resazurin-based assay. *T. brucei* bloodstream-form cells (panels A and C) or human fibroblasts (panels B and D) were diluted across a range of densities on 96-well plates. Resazurin was added immediately in the case of *T. brucei* or the following day for human fibroblasts. Fluorescence signal from *T. brucei* plates was proportional to cell density at various time points (panel A, open circles 2 h; closed circles 3 h; open squares 4 h; closed squares 5 h) and linear with respect to time at various cell densities (panel C, open circles 1 × 10^6^ cells per well; closed circles 5 × 10^5^ cells per well; open squares 2.5 × 10^5^ cells per well; closed squares 1.25 × 10^5^ cells per well). Fluorescence signal from MRC5 plates was proportional to cell density at various time points (panel B, open circles 1 h; closed circles 2 h; open squares 3 h; closed squares 4 h; open triangles 5 h; closed triangles 6 h) and linear with respect to time at various cell densities (panel D, open circles 1 × 10^4^ cells per well; closed circles 1.5 × 10^4^ cells per well; open squares 2 × 10^4^ cells per well; closed squares 2.5 × 10^4^ cells per well; open triangles 3 × 10^4^ cells per well).

AlamarBlue^®^ has been used previously as an *in vitro* viability assay for *T. brucei* [13]. This proprietary reagent contains resazurin [14], a dye which is metabolically reduced in cells to the highly fluorescent product resorufin. Using the less expensive and widely available reagent resazurin, the linearity and dynamic range of the assay was determined for parasite and human fibroblasts. To determine the dynamic range of the assay, bloodstream forms of *T. brucei* were incubated at 37 °C in culture medium at a range of cell densities containing 440 μM resazurin and fluorescence measured at intervals. For each time point fluorescence was proportional to cell density with linear correlation coefficients (*r*^2^) >0.997 (Fig. 1A). Likewise, fluorescence increased linearly with time at any cell density (Fig. 1C, *r*^2^ > 0.996). Thus, fluorescence is proportional to cell density up to 2 × 10^5^ cells per well within a 2–5 h incubation period. Similar results were obtained for human fibroblasts (Fig. 1B and D) up to 3 × 10^4^ cells per well within a 2–6 h incubation. In further assays, final cell densities and resazurin-incubation times did not exceed these ranges.

### 3.2 DMSO tolerance of the resazurin cell viability assays

Test compounds were supplied as 10 mM stocks dissolved in DMSO and so it was necessary to determine the tolerance of the assays for this solvent. Both cell lines showed a similar sensitivity to DMSO with IC_50_ values of 0.80 ± 0.06 and 1.04 ± 0.08% (v/v) for *T. brucei* and human fibroblasts, respectively (data not shown). DMSO (0.5%) was selected as an acceptable compromise allowing compound addition up to 50 μM with an acceptable attenuation of fluorescence of 20% for the *T. brucei* assay and 15% for the MRC5 assay. All subsequent assays contained 0.5% DMSO.

### 3.3 Sensitivity to standard drugs

**Fig. 2:**
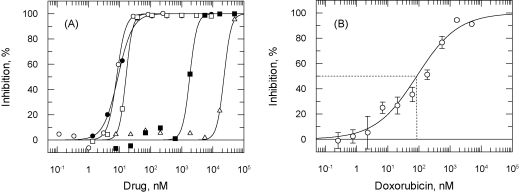
Response of *T. brucei* and human fibroblasts to standard drugs. *T. brucei*, 200 cells per well (panel A) or MRC5, 2000 cells per well (panel B) were incubated for 3 days with a range of inhibitor concentrations. Resazurin was added approximately 4 h before determining percentage inhibition of the fluorescent signal compared to controls minus inhibitor. The EC_50_ values determined were as follows: pentamidine (open circles) 8.10 ± 0.54 nM; melarsoprol (closed circles) 7.52 ± 0.28 nM; suramin (open squares) 20.1 ± 1.1 nM; nifurtimox (closed squares) 1.81 ± 0.13 μM; eflornithine (open triangles) 22.4 ± 1.1 μM. Doxorubicin yielded an EC_50_ of 85.9 ± 38.2 nM.

Five drugs currently used in treatment of HAT were tested for growth inhibition (Fig. 2A). The most potent of these were pentamidine, suramin and melarsoprol, with EC_50_ values in the low nanomolar range, similar to those published for *T. b. rhodesiense* and *T. b. gambiense* by Raz et al. [13] and for various *T. b. gambiense* stocks by Likeufack et al. [15]. Eflornithine was the least effective drug (EC_50_ 22.4 ± 1.1 μM). Since eflornithine induces physiological activation of mitochondrial metabolism [16], it could potentially yield erroneous results due to increased reduction of resazurin in drug-treated cells. However, this does not appear to be the case as direct counting of cell growth returned a similar EC_50_ of 17.1 ± 0.1 μM. Nifurtimox was intermediate in potency (EC_50_ 1.8 μM) in good agreement with previous reports [17,18]. These results provide further validation of resazurin as a suitable substitute for alamarBlue^®^ reagent. Pentamidine isethionate was chosen for use as a ‘per-plate’ control for all future assays. Similarly, the anticancer compound doxorubicin [19] was used as a control for MRC5 assays (Fig. 2B).

### 3.4 Screening of the LOPAC collection

Initially, the LOPAC collection of pharmacologically active compounds was screened against *T. brucei* at a fixed concentration of 1 μM on two independent occasions. This identified 52 compounds producing a mean signal of less than 50% of the control on at least one occasion: an initial hit rate of 4.3%. Hits were analysed by LC–MS confirming a purity of at least 95% for all compounds (data not shown).

Triplicate independent EC_50_ determinations were carried out for each of these hits against *T. brucei*. The resulting EC_50_ values for each hit were in good agreement with coefficients of variation typically less than 20%. Means weighted to standard error for each compound were determined and at this point 19 hits with EC_50_ values of greater than 1 μM were rejected (Table 1), leaving 33 confirmed submicromolar EC_50_ hits against *T. brucei* (Table 2).

Selective activity against the parasite is an essential feature of drug leads [20]. We therefore determined EC_50_ values of our hits against MRC5 cells, a human embryonic lung cell line used in similar previous studies [21]. Coefficients of variation between the resulting 4 determinations were typically less than 40%. Means weighted to standard error were determined and used to calculate the fold-specificity for *T. brucei* over human fibroblasts (Table 2). Two known trypanocidal drugs, pentamidine and suramin, which happen to be present in the LOPAC library returned specificities of 3500- and >700-fold, respectively. The highest specificity of the remaining hits (270-fold) was demonstrated by (+)-U50,488, with an EC_50_ of 59 nM. The mixture of enantiomers, (±)-U50,488, showed a similar specificity, but only half the potency of the 1*R*,2*R trans* enantiomer, suggesting that the growth inhibition is largely due to (+)-U50,488. Only two other hits returned specificities greater than 100-fold: dequalinium dichloride, an broad-range antimicrobial known to act against plasmodium in mice [22] and oligomycin A, a macrolide antibiotic that inhibits membrane associated mitochondrial ATPase [23]. Only one compound (vincristine sulfate, an inhibitor of microtubule assembly [24]) demonstrated greater specificity for human fibroblasts compared to *T. brucei*. Among the other potent, but less selective screening hits were compound classes with activity against protein kinases, topoisomerases, tubulin, as well as DNA and energy metabolism. The average *Z*′ scores throughout the testing was 0.66 for *T. brucei* assays and 0.57 for MRC5 assays, values characteristic of high quality cell-based screening assays.

### 3.5 Confirmation of inhibition by (+)-U50,488 and other opioid receptor ligands

**Fig. 3:**
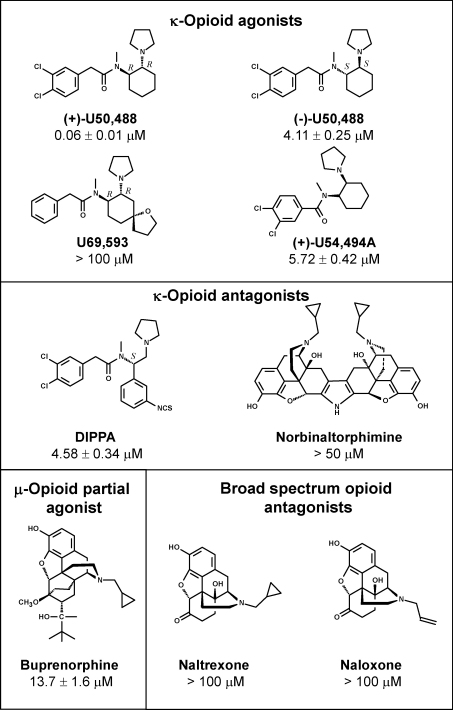
Structure–activity relationships of opioid receptor ligands against *T. brucei*. These compounds were screened for potency against *T. brucei*. EC_50_ values are recorded as the weighted mean of 3 determinations. Norbinaltorphimine was tested at a maximum concentration of 50 μM due to solubility issues.

A sample of (+)-U50,488 and eight other compounds known to act on μ- or κ-opioid receptors were purchased for EC_50_ determination. The mean weighted to standard error was 58.9 nM for the authentic (+)-U50,488 sample compared to the 60 nM value found during the library screening. Of the other opioid receptor ligands with structural similarity to (+)-U50,488, three displayed EC_50_ values ∼5 μM (Fig. 3) and one (U69593) was inactive. Of the compounds showing structural similarity to morphine, only buprenorphine showed weak activity. Thus, the original (+)-U50,488 hit had the highest potency of this group of compounds and was 70 times more potent than its *trans* enantiomer, (−)-U50,488.

### 3.6 κ-Opioid receptor homology searching

Global alignments of the amino acid sequences of the κ-opioid receptor from sheep, zebrafish, rough skinned newt, mouse and guinea pig return similarity scores of 60–99%. TBLASTN searching of the *T. brucei* GeneDB with these query sequences revealed several predicted genes with only short stretches of alignment. Global alignments of these predicted genes with the known receptor sequences returned similarity scores of only 9–19%.

### 3.7 Effect of (+)-U50,488 on *T. brucei in vivo*

**Fig. 4:**
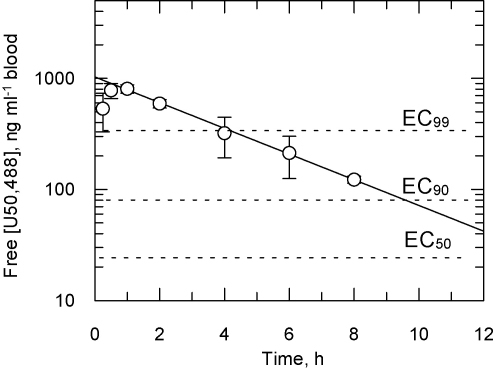
Pharmacokinetic properties of (+)-U50,488 in mice. Data show the mean and standard error for the free blood concentration in 3 mice following a single oral dose at 150 mg kg^−1^. The calculated half-life is 2.60 ± 0.04 h. The dashed lines indicate the EC_50_ obtained *in vitro* against *T. brucei* and the predicted EC_90_ and EC_99_ values for growth inhibition.

Pharmacokinetic studies on (+)-U50,488 in uninfected mice were performed. The compound is not tightly bound to mouse plasma proteins (fraction unbound is 0.69) and is well tolerated when administered at 150 mg kg^−1^ via the oral route. The brain to blood ratio was 8.2, an excellent property for a stage 2 candidate HAT drug. The peak free blood concentration of (+)-U50,488 was 800 ng ml^−1^ 1 h after dosing with a half-life of 2.6 ± 0.04 h (Fig. 4). Based on these findings, 3 mice were infected with *T. brucei* and dosed orally at 150 mg kg^−1^ every 4 h for 32 h to ensure that the free blood level remained above the EC_99_. However, this regimen was not curative, with only one mouse showing a transient reduction in parasitaemia followed by relapse, in sharp contrast to our recent studies on inhibitors of *N*-myristoyltransferase [25] and nitroaromatic drugs [18].

### 3.8 Effect of (+)-U50,488 and HAT drugs on *T. brucei* culture growth

**Fig. 5:**
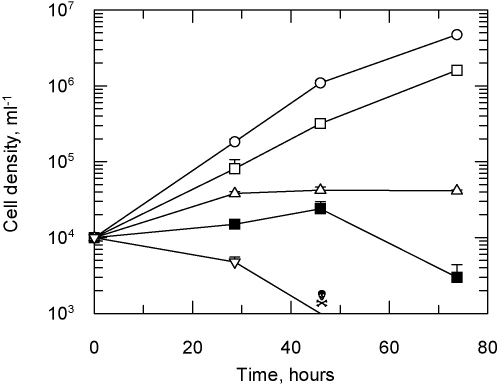
Effect of inhibitors on *T. brucei* growth *in vitro*. *T. brucei* were grown in 10-ml cultures in the presence of the inhibitors at 10 times (open symbols) and 500 times (closed symbols) their EC_50_ values. Cells were counted using a haemocytometer over a 3-day period. Values are the mean and standard deviation of three flasks and are representative of three independent experiments. No drug, circles; (+)-U50,488, squares; eflornithine, triangles; pentamidine, inverted triangle. Cell counts below the limits of detection are indicated by a skull and crossbones symbol.

To examine the cause of the poor trypanocidal activity *in vivo*, (+)-U50,488 was compared with the known HAT drugs pentamidine and eflornithine for their ability to kill or slow the growth of *T. brucei* cells in culture (Fig. 5). Exposure to pentamidine (10 × EC_50_) caused the cell density to drop below the limit of detection (1 × 10^3^ cells ml^−1^) by 40 h with no viable cells visible by microscopy, indicating a cytocidal effect. Eflornithine arrested cell growth after 30 h and cell density did not change thereafter, indicating a cytostatic effect. In contrast, exposure to (+)-U50,488 at 10 × EC_50_ only had a minor effect on growth, reducing the doubling time from 6.8 to 9.7 h. Further inspection of the EC_50_ curves suggested that this unexpected result might be attributed to a low Hill slope for this compound. Based on the EC_50_ values and Hill slopes for pentamidine and eflornithine (8 nM, *s* = 3.1 and 22.4 μM, *s* = 4.0, respectively) EC_99_ values of 37 nM and 77 μM can be calculated, values which are less than 10 times EC_50_. In contrast, (+)-U50,488 (EC_50_ 60 nM, *s* = 1.8) has a predicted EC_99_ value of 740 nM, which is greater than 10 times the EC_50_. Thus, the observed partial inhibition of growth is consistent with exposure to this concentration of drug. In a series of additional experiments the effect on growth of 50, 100 and 500 times the EC_50_ for (+)-U50,488 was examined. These concentrations showed a dose dependent reduction in growth rate, but only at the highest drug concentration (30 μM, 500 times EC_50_) was it possible to achieve a cytocidal effect (Fig. 5, closed squares).

## 4 Discussion

Our experiments establish that resazurin can be used as a cheaper substitute for the alamarBlue^®^ formulation for growth inhibition assays for bloodstream-form *T. brucei* or human fibroblasts. However, this assay method does not necessarily indicate cell death. Inhibitors could be cytocidal, cytostatic, or interfering with the reduction of resazurin without effect on cell growth. Lead compounds must therefore be assessed for cytotoxicity by alternative means such as direct cell counting of viable parasites (Fig. 5) or a ‘live–dead assay’ [26]. The HMI9-T medium used in this assay has been shown to mask inhibition of certain metabolic pathways such as folate metabolism and thymidylate biosynthesis [27] and consequently these inhibitors are unlikely to be identified with these culture conditions. Given this proviso, our results indicate that this semi-automated medium-throughput screening method against *T. brucei* is reproducible and robust. With further automation and miniaturisation it could be used to screen much larger compound collections and other special focussed sets available in our Drug Discovery Unit [28].

The drugs currently used to treat HAT show a wide range of potencies in this assay, ranging from 8 nM for pentamidine and melarsoprol to 1.8 μM for nifurtimox and 22 μM for eflornithine. Despite many current drug-discovery paradigms being driven by potency – at least at the early stages of lead identification – the example of eflornithine demonstrates that lower potency compounds may still emerge as effective treatments. Any lead offering improvement on either the mode of administration or cost of eflornithine regimen would be an advance in the treatment of HAT.

The EC_50_ values determined by Likeufack et al. against *T. b. gambiense* vary by a maximum of 4.5-fold between stocks [15], and the values we report against *T. b. brucei* fall within these ranges. The same is true for the values reported by Raz et al. [13] for *T. b. rhodesiense*. However, although *T. b. brucei*, which is non-pathogenic to humans, is a suitable model for HAT, it is important to test advanced lead compounds against these clinically relevant subspecies.

The 33 inhibitors of *T. brucei* with nanomolar potency identified from the LOPAC collection included pentamidine and suramin which acted as internal controls. The minor differences in EC_50_ values between the library screening and our testing of standard compounds could be due to higher purity in the original samples purchased and evaporation in the smaller volumes of the library samples. Original hits that were rejected at the 1 μM cut-off include several compounds that have known pharmacological activity in animal models. Testing these against rodent models of HAT at the already-established maximum tolerated doses could identify additional promising leads.

Six out of the 33 hits with EC_50_ values less than 1 μM showed more than 100-fold selectivity for *T. brucei*. Pentamidine and suramin were the most selective compounds identified, illustrating the target selectivity (>1000-fold) that should be aimed for at the final stages of lead development. The most selective compounds previously unknown to inhibit *T. brucei* were from isomers of U50,488, a known agonist of the κ-opioid receptor [29]. This compound shows good brain penetration and therefore potentially able to access a late-stage HAT infection, a key feature specified by the current target product profile from WHO.

From the limited number of commercially available opioid receptor ligands, (+)-U50,488 was the most potent (Fig. 4). Other compounds showed either weak or no activity. The complete lack of activity of norbinaltorphimine against *T. rucei* suggests that possible CNS side effects of a treatment based on (+)-U50,488 could be reduced by co-administration of a κ-opioid antagonist. Whilst the (-)-U50,488 isomer is a stronger agonist of the κ-opioid receptor than the (+)-U50,488 isomer, the latter is almost 70 times more effective against *T. brucei*. κ-Opioid receptors typically share high similarity at the amino acid level across species as indicated by our homology searching. However, consistent with the poor activity of other opioid receptor ligands, we found no convincing homologue of the receptor in the *T. brucei* genome. An opioid receptor agonist (endomorphin-1; YPWF-NH_2_) has been shown to inhibit a drug-metabolite transporter from the malaria parasite *Plasmodium falciparum* [30]. Mutations in this transporter confer resistance to chloroquine, but unfortunately the physiological substrates are not known [31]. Moreover, no convincing homologues could be identified in the *T. brucei* genome database. Thus, the target for U50,488 remains to be identified in *T. brucei*.

The *in vivo* and *in vitro* experiments indicate that further optimization of (+)-U50,488 is required to achieve efficacy. Although the compound has good pharmacological properties, including high CNS penetration, it was not curative using a therapeutic schedule which was predicted to maintain free blood exposure above the predicted EC_99_. The reason for this is not entirely clear. The *in vitro* studies demonstrated that the compound is cytostatic at 100 times the EC_50_, like eflornithine, rather than cytocidal, like pentamidine. Thus, continuous exposure above the minimum inhibitory concentration may be required to allow sufficient time for the immune system to eliminate non-dividing parasites, as is the case with eflornithine. Alternatively, differences in the physiological environment *in vivo* and *in vitro* may be responsible for the lack of efficacy. Further studies on the mode of action of (+)-U50,488 would be informative. Although a cytocidal effect was observed at high concentrations (30 μM, 500 times EC_50_), this could be due to additional off-target effects and, in any case, is above the maximum free blood concentration achievable under the dosing schedule used here.

Future work will involve further medicinal chemistry to explore structure–activity relationships, improve potency and the Hill slope. It remains to be seen whether the cytostatic effect of U50,488 is a function of the target(s) or the inhibitor itself. Identifying the intracellular target of U50,488 could provide a new avenue for drug discovery efforts against *T. brucei*.

## Figures and Tables

**Table 1:** Initial submicromolar hits from the LOPAC library returning EC_50_ vs. *T. brucei* greater than 1 μM.

Compound	EC_50_ vs. *T. brucei* (μM)
Parthenolide	1.06 ± 0.08
CB 1954^a^	1.08 ± 0.09
Cyclosporin A	1.14 ± 0.17
Bromoacetyl alprenolol menthane	1.26 ± 0.14
Indirubin-3′-oxime	1.28 ± 0.09
Dipropyldopamine hydrobromide	1.29 ± 0.06
SB 224289 hydrochloride^b^	1.35 ± 0.28
*R*(−)-Propylnorapomorphine hydrochloride	1.52 ± 0.21
1,3-PBIT dihydrobromide^c^	1.58 ± 0.08
Caffeic acid phenethyl ester	1.64 ± 0.13
Kenpaullone	1.86 ± 0.27
CGS-15943^d^	1.97 ± 0.21
A-77636 hydrochloride^e^	2.21 ± 0.15
Retinoic acid p-hydroxyanilide	2.23 ± 0.27
6-Hydroxymelatonin	3.31 ± 0.23
*S*-(4-Nitrobenzyl)-6-thioinosine	3.78 ± 0.55
*S*-(*p*-Azidophenacyl)glutathione	>10
Dephostatin	>10
2′,3′-Dideoxycytidine	>10

a 5-(1-Aziridinyl)-2,4-dinitrobenzamide.

b 1′-Methyl-5-([2′-methyl-4′-(5-methyl-1,2,4-oxadiazol-3-yl)biphenyl-4-yl] carbonyl)-2,3,6,7-tetrahydro-spiro(furo[2,3-f]indole-3,4′-piperidine).

c Phenylene-1,3-bis(ethane-2-isothiourea) dihydrobromide.

d 9-Chloro-2-(2-furyl)[1,2,4]triazolo[1,5-c]quinazolin-5-amine.

e (−)-(1*R*,3*S*)-3-Adamantyl-1-(aminomethyl)-3,4-dihydro-5,6-dihydroxy-1H-2-benzopyran hydrochloride hydrate.

**Table 2:** Submicromolar hits against *T. brucei* ranked by selectivity vs. MRC5 cells.

Compound	EC_50_ vs. *T. brucei* (nM)	EC_50_ vs. MRC5 (nM)	Selectivity
Pentamidine isethionate	3.7 ± 0.10	13 000 ± 6800	3400
Suramin hexasodium	68 ± 3.5	>50 000	>710
(+)-*trans*-(1*R*,2*R*)-U-50488 hydrochloride	59 ± 2.2	16 000 ± 4900	270
(±) *trans*-U-50488 methanesulfonate	113 ± 7.6	26 000 ± 8700	230
Dequalinium dichloride	43 ± 8.8	6100 ± 1500	140
Oligomycin A	150 ± 3.1	18 000 ± 2600	120
Mitoxantrone	2.4 ± 0.20	110 ± 37	46
8-(4-Chlorophenylthio)-cAMP sodium	400 ± 54	18 000 ± 12 000	44
Chelerythrine chloride	59 ± 5.7	2500 ± 480	42
Pimozide	340 ± 19	14 000 ± 3300	41
8-Cyclopentyl-1,3-dipropylxanthine	530 ± 53	17 000 ± 7900	31
4-Chloromercuribenzoic acid	690 ± 85	19 000 ± 3000	27
Dihydroergocristine methanesulfonate	890 ± 57	20 000 ± 770	23
Dequalinium analogue, C-14 linker	70.0 ± 4.6	1000 290	14
Calmidazolium chloride	510 ± 66	6600 ± 750	13
Purvalanol A	820 ± 55	10 000 ± 3200	12
Beta-Lapachone	850 ± 130	8600 ± 2900	10
Vinblastine sulfate salt	94 ± 6.0	780 ± 910	8.3
CGP-74514A^a^	210 ± 8.3	1500 ± 620	7.1
Sanguinarine chloride	240 ± 17	1400 ± 350	5.8
Diphenyleneiodonium chloride	120 ± 7.6	700 ± 870	5.7
Sphingosine	820 ± 78	4600 ± 2500	5.7
Quinacrine dihydrochloride	380 ± 13	1900 ± 440	5.0
SKF 89145 hydrobromide^b^	610 ± 44	2900 ± 200	4.7
Calcimycin	180 ± 13	750 ± 140	4.2
Idarubicin	20 ± 1.5	78 ± 29	3.9
Emetine dihydrochloride hydrate	7.9 ± 0.53	30 ± 3.1	3.8
Ellipticine	460 ± 45	1700 ± 620	3.7
Mevastatin	940 ± 50	2000 ± 360	2.2
Taxol	3.3 ± 0.071	6.6 ± 9.8	2
(*S*)-(+)-Camptothecin	530 ± 13	800 ± 99	1.5
Alsterpaullone	400 ± 40	510 ± 170	1.3
Vincristine sulfate	30 ± 1.0	0.60 ± 1.0	0.02

a *N*^2^-(cis-2-Aminocyclohexyl)-*N*^6^-(3-chlorophenyl)-9-ethyl-9H-purine-2,6-diamine HCl.

b 4-(3,4-Dihydroxyphenyl)-6-methyl-4,5,6,7-tetrahydrothieno[2,3-c]pyridine HBr.
